# Both T cell priming in lymph node and CXCR3-dependent migration are the key events for predicting the response of atezolizumab

**DOI:** 10.1038/s41598-021-93113-y

**Published:** 2021-07-06

**Authors:** Toshiki Iwai, Masamichi Sugimoto, Namrata S. Patil, Daniel Bower, Miho Suzuki, Chie Kato, Keigo Yorozu, Mitsue Kurasawa, David S. Shames, Osamu Kondoh

**Affiliations:** 1 Product Research Department, Kamakura Research Laboratories, Chugai Pharmaceutical Co., Ltd., 200 Kajiwara, Kamakura, Kanagawa 247-8530 Japan; 2 Oncology Biomarker Development, Genentech, Inc., South San Francisco, USA; 3 Research Division, Kamakura Research Laboratories, Chugai Pharmaceutical Co., Ltd., Kamakura, Kanagawa Japan

**Keywords:** Cancer immunotherapy, Lung cancer, Tumour immunology

## Abstract

Anti-PD-L1 antibodies benefit many cancer patients, even those with “non-inflamed tumor”. Determining which patients will benefit remains an important clinical goal. In a non-inflamed tumor mouse model, we found that PD-L1 was highly expressed on antigen-presenting cells (APCs) especially on CD103^+^ CD11c^+^ dendritic cells in tumor-draining lymph nodes (dLNs), suppressing T-cell priming by APCs. In this model, anti-PD-L1 antibodies enhanced T-cell priming and increased CXCR3^+^ activated T-cells in dLNs, which was followed by the trafficking of T-cells to tumors in response to CXCR3 ligands. As predictive biomarker, each APCs-related gene expression (AP score) alone or T-cells trafficking-related chemokine gene expression (T score) alone were still less than perfect among the 17 mouse models examined. However a combining score of AP score and T score (AP/T score) precisely identified anti-PD-L1-sensitive tumors. In the phase 3 trial of atezolizumab vs docetaxel in advanced NSCLC patients (OAK), the AP/T score could identify atezolizumab-treated NSCLC patients who achieved significant improvement in overall survival. This biomarker concept would be a clinically valuable for prediction of anti-PD-L1 antibody efficacy.

## Introduction

Programmed Death Ligand 1 (PD-L1) plays a major role in suppressing the host’s antitumor immune response^1^. Interaction between PD-L1 present on cancer cells or tumor-infiltrating immune cells and its receptors PD-1 and B7-1 (also known as CD80) on T cells delivers a signal that inhibits the activation of T cells^2^. Recently, anti-PD-1 and anti-PD-L1 antibodies have been developed to counter this and reinvigorate the host’s antitumor immune response.

Anti-PD-1 and anti-PD-L1 antibodies generally show a higher clinical benefit specifically in patients with high intratumoral PD-L1 expression. Interestingly, however, the phase 3 OAK trial showed an overall survival (OS) advantage for atezolizumab (an anti-PD-L1 antibody) versus docetaxel even in the subgroup with immunohistochemically low or undetectable PD-L1 on tumor cells and immune cells (TC0/IC0)^3^. A similar OS benefit was shown in patients with low PD-L1 levels as determined by analysis of PD-L1 gene expression in tumor tissues^3^. Therefore, the value of intratumoral PD-L1 expression in predicting a patient’s response to such treatment is still less than perfect. Therefore, determining which patients derive benefit from anti-PD-L1 therapy remains an important clinical question.

PD-L1 is also reported to be expressed on dendritic cells (DCs) from lymph nodes draining the retroperitoneum of patients with cancer^4^, and PD-1 is reported to be highly expressed on T cells not only within tumor tissue but also in lung tumor-draining lymph nodes^5,6^. In addition, PD-L1 competes with CD28 for B7-1 binding on antigen-presenting cells, suppressing T cell priming by outcompeting signaling through CD28/B7-1^7^. However, the role that T cell priming in lymph nodes plays in the mechanism of action for anti-PD-L1 therapy has not yet been clarified.

In this study, we used syngeneic mouse tumor models to investigate the mechanism of action by which PD-L1-negative tumors responded to treatment with an anti-PD-L1 monoclonal antibody (mAb), and we investigated a novel biomarker based on the inferred mechanism of action.

## Results

### Antitumor activity of anti-PD-L1 mAb classified by PD-L1 gene expression in tumor tissues

**Figure 1:**
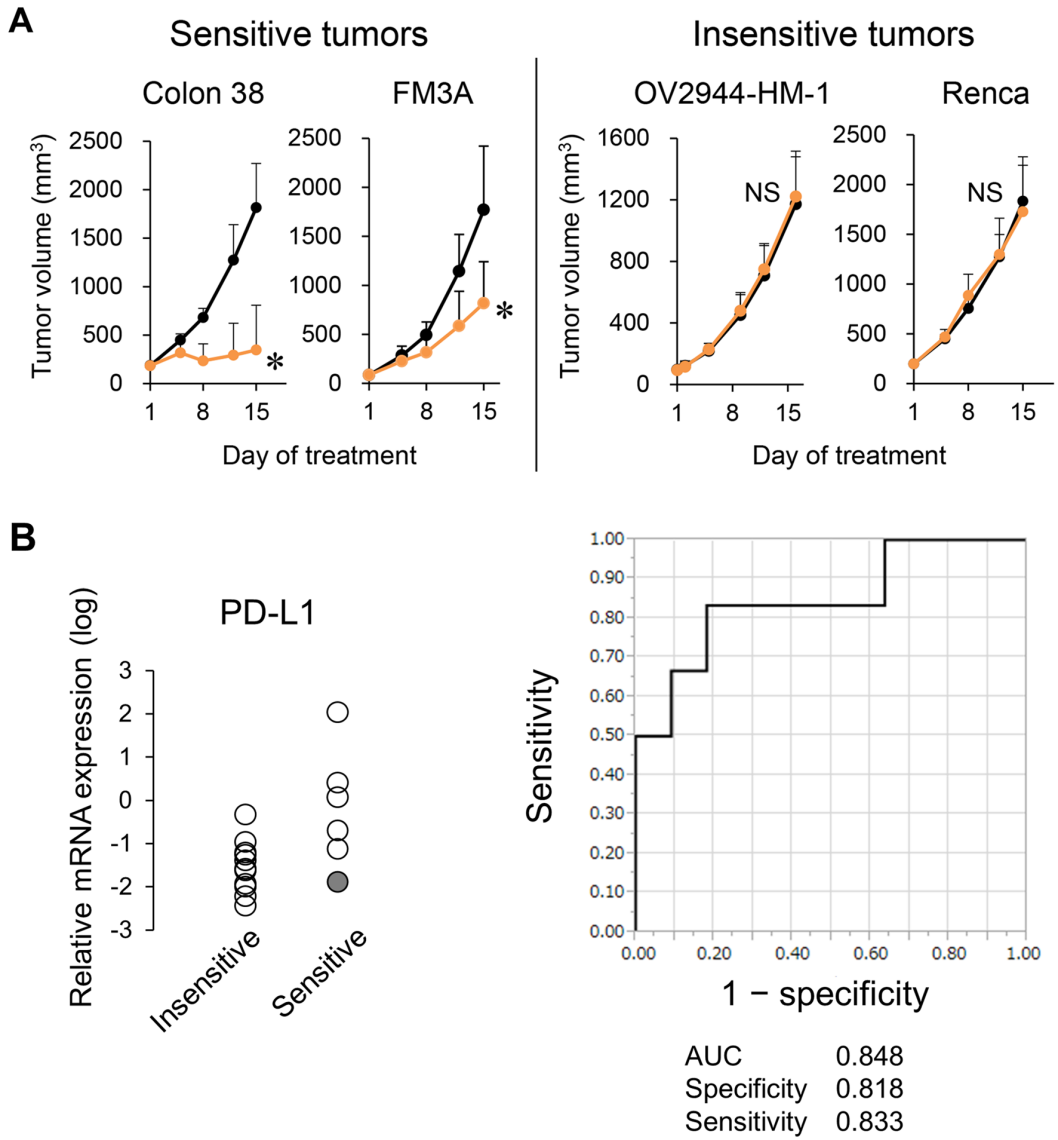
Relation between antitumor activity of anti-PD-L1 mAb and PD-L1 expression. (**A**) Representative tumor growth curves: mice bearing tumors were randomly divided into groups. Anti-mouse PD-L1 mAb or Rat IgG was administered intraperitoneally to the mice at a dose of 10 mg/kg three times a week. Black, control; orange, anti-PD-L1 mAb 10 mg/kg. Data are shown as the mean + SD (*n* = 5–15/group). Statistical analysis used Wilcoxon rank sum test. *P < 0.05; *NS* not significant. (**B**) PD-L1 mRNA expression in tumor tissues at baseline insensitive or sensitive to anti-PD-L1 mAb (left), and ROC curve analysis (right). Shaded circle indicates results for the FM3A model.

We examined the antitumor activity of the anti-PD-L1 mAb in 17 murine tumor models. The anti-PD-L1 mAb showed significant antitumor activity in six of the tumor models. However in 11 tumor models, the anti-PD-L1 mAb did not show significant antitumor activity (Supplementary Table S1, Fig. 1A).

To examine the ability of intratumoral PD-L1 expression levels to classify tumors as sensitive or insensitive to the anti-PD-L1 mAb, we measured PD-L1 mRNA expression in tumor samples by RNA sequencing and analyzed the ROC curve. The range of PD-L1 mRNA expression levels in the sensitive models was wide and overlapped the range in the insensitive tumor models. The area under the ROC curve (AUC) was 0.848. Specificity and sensitivity at the best cut-off point as indicated by Youden’s index were 0.818 and 0.833, respectively (Fig. 1B).

### Anti-PD-L1 mAb exerted antitumor activity in a PD-L1-negative and immune desert-like tumor model

To examine why intratumoral PD-L1 mRNA expression did not entirely predict the antitumor activity of the anti-PD-L1 mAb in the mouse models, we analyzed the mechanism of action of the anti-PD-L1 mAb in a low-PD-L1 tumor model. As a low-PD-L1 tumor model, we selected the FM3A model which showed the lowest intratumoral PD-L1 mRNA expression among the models classified as being sensitive to the anti-PD-L1 mAb (Fig. 1B).

**Figure 2:**
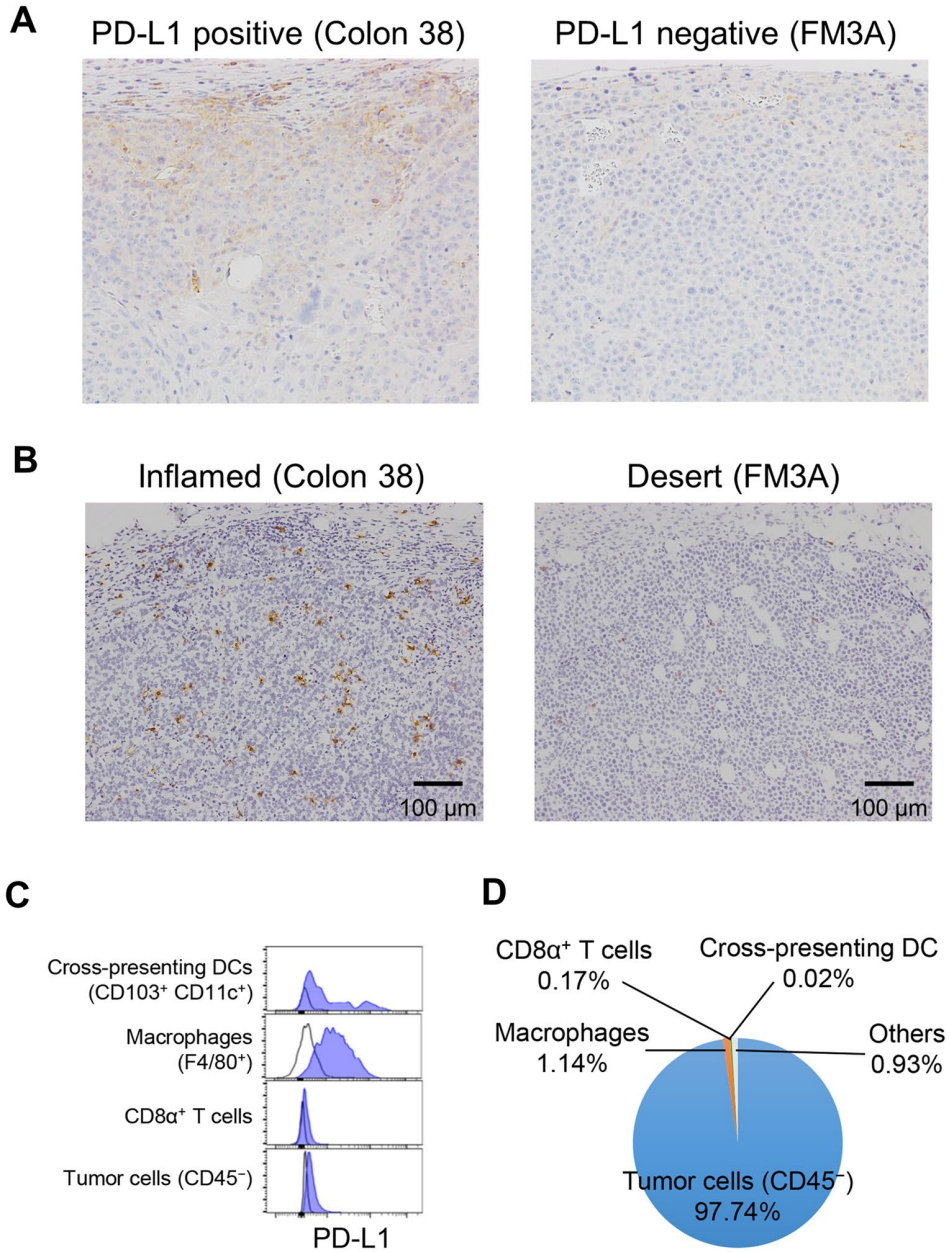
PD-L1 expression and CD8α^+^ infiltration in FM3A tumor model. (**A**) PD-L1 immunohistochemical staining in tumor tissue at baseline in the Colon 38 model (positive control) and FM3A model. (**B**) CD8α immunohistochemical staining in tumor tissue at baseline. (**C**) PD-L1 expression on tumor-infiltrating immune cells and tumor cells in FM3A tumors. (**D**) Distribution of cells in FM3A tumors. Cells were determined by flow cytometric analysis.

First, we characterized the FM3A tumors histochemically. PD-L1 immunohistochemical staining showed that nearly all of the cells in the tumor tissue were negative under conditions in which Colon 38, another sensitive tumor, showed partial positive staining (Fig. 2A). CD8α immunohistochemical staining showed that CD8α^+^ T cells had infiltrated into Colon 38 tumors but there were very few CD8α^+^ T cells in the FM3A tumors (Fig. 2B).

Next, using flow cytometric analysis, we found that F4/80^+^ cells in FM3A tumors expressed PD-L1 but were found in only small numbers in the tumor tissue (Fig. 2C,D). Tumor cells (CD45^−^ cells), which expressed almost no PD-L1, constituted most of the tumor tissue (Fig. 2C,D). These results show that FM3A tumors have a PD-L1-negative and immune desert-like phenotype.

### Anti-PD-L1 mAb enhanced T cell priming that had already occurred in lymph nodes

**Figure 3:**
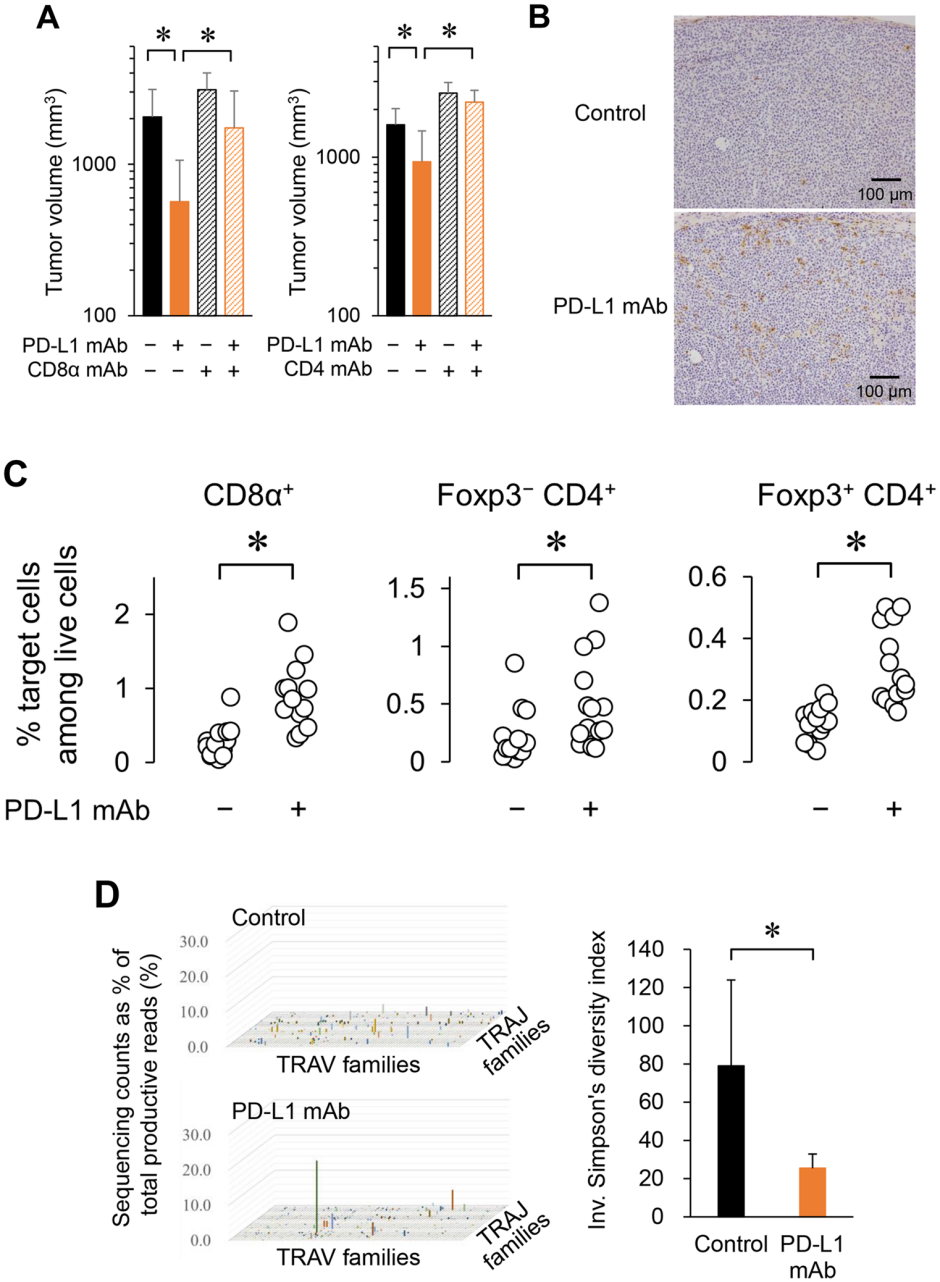
Anti-PD-L1 mAb increases specific T cells in tumor, resulting in T cell-dependent antitumor activity in the PD-L1-negative tumor model. (**A**) Tumor volume of mice treated with anti-PD-L1 mAb along with CD8α mAb or CD4 mAb in the FM3A tumor model on Day 19 or Day14, respectively (*n* = 7/group). Data are shown as the mean + SD. Statistical analysis used Wilcoxon rank sum test and the Holm–Bonferroni method. *P < 0.05. (**B**) Infiltration of CD8α^+^ T cells into tumors was determined by CD8α immunohistochemical staining in tumor tissue after anti-PD-L1 mAb treatment (Day 19). (**C**) Percentage of T cells in tumor after anti-PD-L1 mAb treatment (Day 19) (*n* = 14/group). Cells were determined by flow cytometric analysis. Statistical analysis used Wilcoxon rank sum test. *P < 0.05. (**D**) Percentage of TCRα variable (TRAV) and TCRα joining (TRAJ) gene segments in tumor after anti-PD-L1 mAb treatment (Day 19). Next-generation sequencing was performed with unbiased TCR repertoire analysis technology (Repertoire Genesis). The inverse of Simpson’s diversity index was used to examine diversity based on the clonal dominance of each TCR clonotype (*n* = 3–5/group). Data are shown as the mean + SD.

We then investigated whether the anti-tumor activity of the anti-PD-L1 mAb in the FM3A tumor model was dependent on T cells. Depletion of CD8α^+^ or CD4^+^ T cells abolished the antitumor activity of the anti-PD-L1 mAb (Fig. 3A). The anti-PD-L1 mAb significantly increased the population of T cells including CD8α^+^ cells, Foxp3^−^CD4^+^ cells, and Foxp3^+^CD4^+^ cells in the tumors (Fig. 3B,C). To examine the levels of T cell diversity in tumors treated with the anti-PD-L1 mAb, we evaluated the diversity of T cell receptor (TCR) repertoires by using Simpson’s diversity index. The variation of TCRα variable and TCRα joining gene segments in the anti-PD-L1 mAb-treated tumors was smaller than that in control tumors, and only specific elements of the TCRα repertoire increased in the anti-PD-L1 mAb-treated tumors (Fig. 3D). The inverse of Simpson’s diversity index was found to be significantly lower in the anti-PD-L1 mAb treatment group compared to the control group (Fig. 3D).

**Figure 4:**
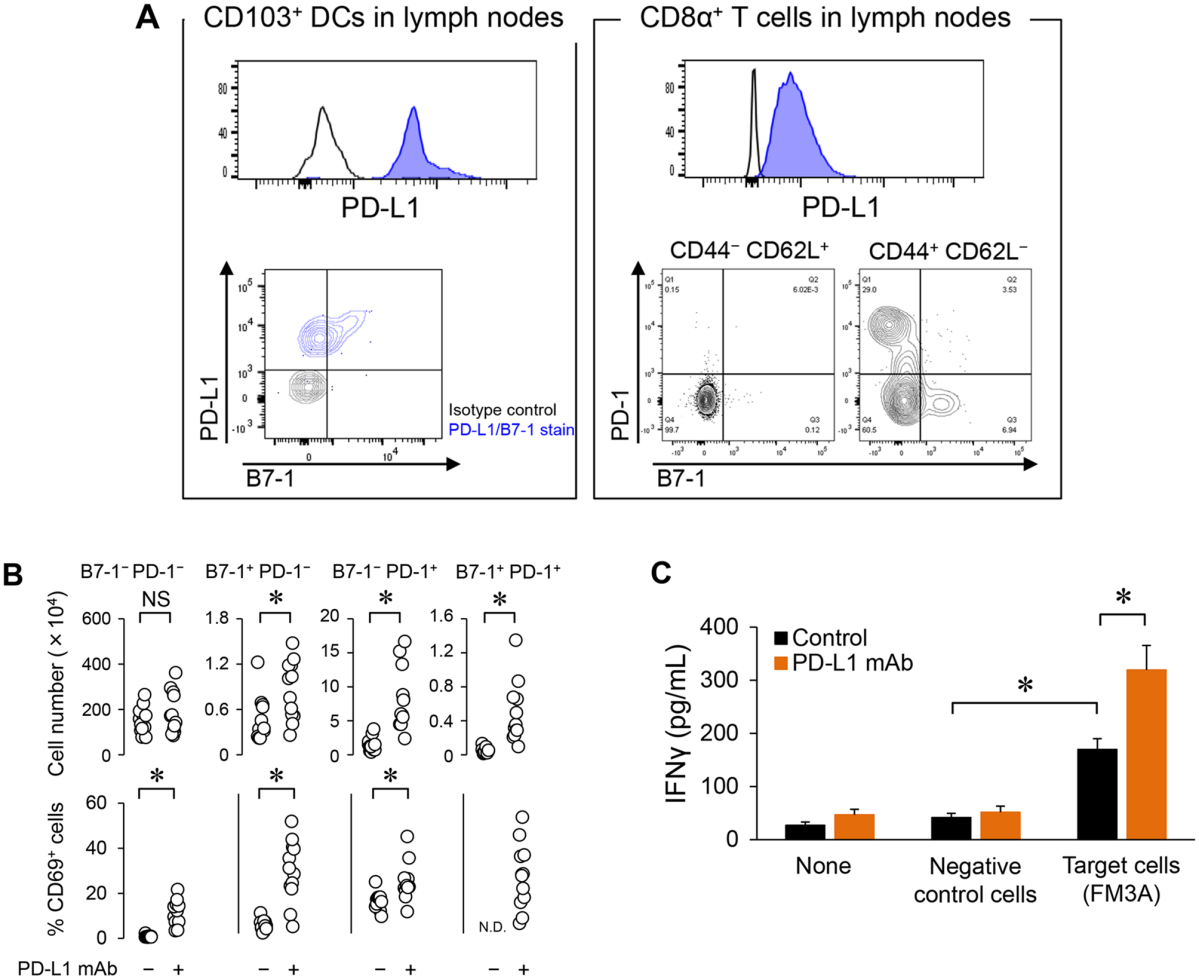
The role of PD-L1 in T cell priming in lymph nodes. (**A**) PD-L1 expression or PD-L1 receptor (PD-1 and B7-1) expression on antigen-presenting dendritic cells (DCs) or CD8α^+^ T cells in tumor-draining lymph nodes at baseline. (**B**) Number of PD-L1 receptor (PD-1 and B7-1)^+^ CD8α^+^ T cells and the frequency of CD69^+^ cells among PD-L1 receptor^+^ CD8α^+^ T cells in tumor-draining lymph nodes after anti-PD-L1 mAb treatment (Day 4) (*n* = 12/group). Statistical analysis used Wilcoxon rank sum test. *P < 0.05; *NS* not significant. (**C**) Secretion of IFNγ after specific stimulation of lymphocytes in tumor-draining lymph nodes by co-culturing with tumor cells (*n* = 3/group). IFNγ was quantified by ELISA. Statistical analysis used Student’s *t* test and the Holm–Bonferroni method. *P < 0.05. Data are shown as the mean + SD.

Because these results suggested that tumor-reactive T cells were enriched by the anti-PD-L1 mAb treatment, we focused on tumor-draining lymph nodes and the role of PD-L1 in T cell priming. First we investigated PD-L1 expression on antigen-presenting DCs in lymph nodes. CD103^+^CD11c^+^ cells expressed PD-L1 in lymph nodes (Fig. 4A left).

Next we analyzed expression of PD-L1 receptors (PD-1 and B7-1) on CD8α^+^ T cells in lymph nodes. Most CD8α^+^ T cells were CD44^−^CD62L^+^ naïve T cells which were PD-1- and B7-1-negative, but some of the CD44^+^CD62L^−^CD8α^+^ effecter T cells were PD-1- and/or B7-1-positive cells (Fig. 4A right). Anti-PD-L1 mAb increased the number of B7-1^+^CD8α^+^ T cells, PD-1^+^CD8α^+^ T cells, and B7-1^+^PD-1^+^CD8α^+^ T cells, and increased the percentage of CD69^+^ cells among B7-1^+^CD8α^+^ T cells, PD-1^+^CD8α^+^ T cells, and B7-1^−^PD-1^−^CD8α^+^ T cells (Fig. 4B). The percentage of CD69^+^ cells among B7-1^+^PD-1^+^CD8α^+^ T cells in the control group could not be determine because of the low number of cells (Fig. 4B).

**Figure 5:**
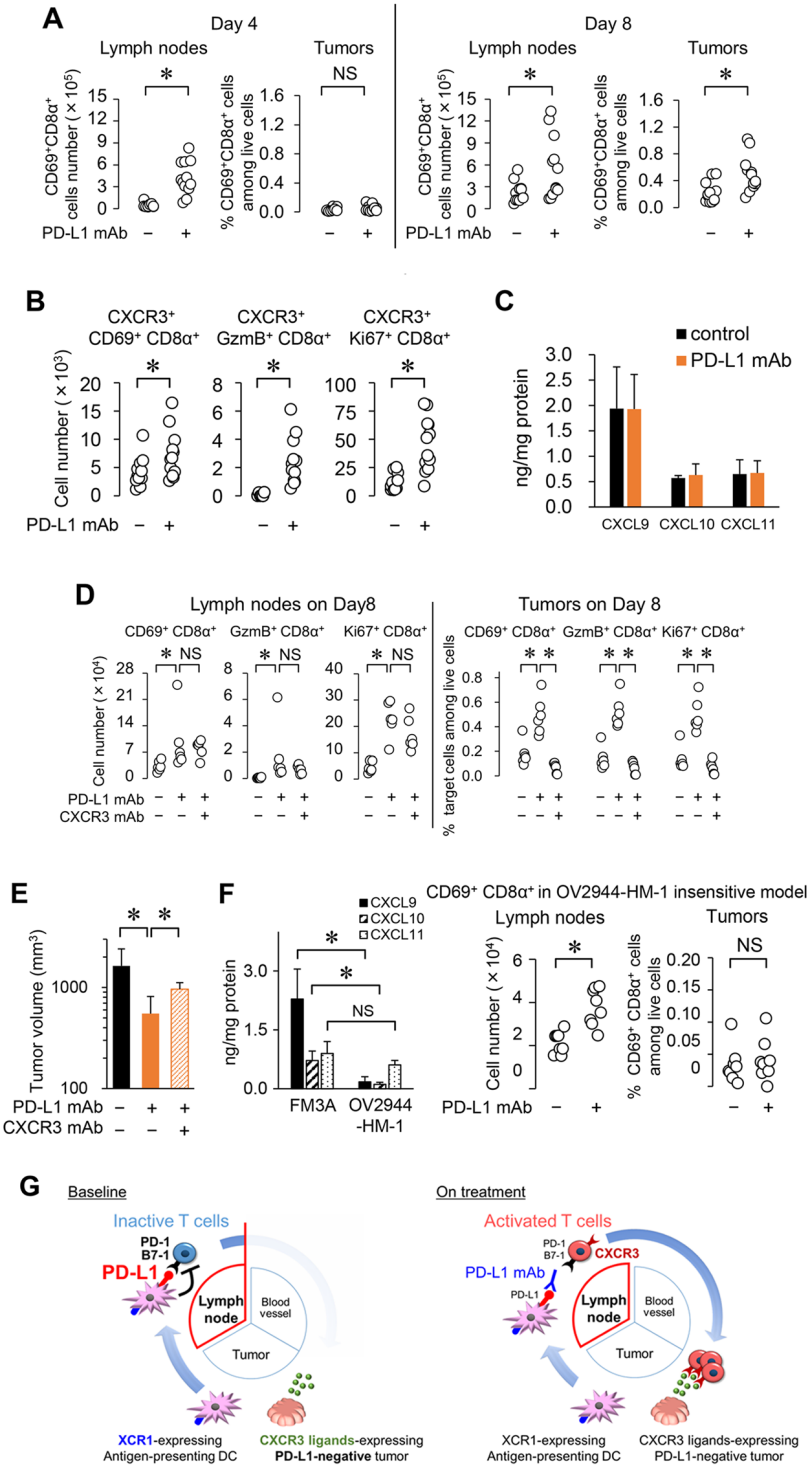
Time lag between the increase in activated CD8α^+^ T cells in lymph nodes and the increase in the tumor is caused by T cell trafficking to a TC0/IC0 tumor. (**A**) Time course of CD8α^+^ T cell activation in tumor-draining lymph nodes and in tumor after anti-PD-L1 mAb treatment in the FM3A model (Day 4, 8) (*n* = 12/group). (**B**) Number of CXCR3^+^ activated CD8α^+^ T cells in lymph nodes after anti-PD-L1 mAb treatment (Day 8) (*n* = 12/group). (**C**) Expression of CXCR3 ligands in tumor (Day 8) (*n* = 6/group). After mice were euthanized by exsanguination on Day 8, tumors were collected and homogenized. (**D**) Number of activated CD8α^+^ T cells in lymph nodes and in tumors after anti-PD-L1 mAb treatment with or without CXCR3 blockade (Day 8) (*n* = 6/group). (**E**) Tumor volume of mice treated with anti-PD-L1 mAb along with anti-CXCR3 mAb in the FM3A tumor model. (Day 18) (*n* = 6/group). (**F**) Expression of CXCR3 ligands in tumors of FM3A and OV2944-HM-1 (left). Number of activated CD8α^+^ T cells in lymph nodes (middle) and in tumors (right) after anti-PD-L1 mAb treatment (Day 8) (*n* = 8/group). Data are shown as the mean + SD. Statistical analysis used Wilcoxon rank sum test and the Holm–Bonferroni method. *P < 0.05; *NS* not significant. (**G**) Hypothesis for mechanism of action of anti-PD-L1 mAb in the PD-L1-negative and immune desert-like tumor model based on the cancer–immunity cycle.

Next we investigated tumor-specific T cell responses during anti-PD-L1 mAb treatment. Using lymphocytes from tumor-draining lymph nodes, we found that IFNγ production was significantly increased in the control group when stimulated with FM3A cells compared to that when stimulated by MBT2 cells (MHC-matched negative control cells), and it was further significantly increased in the anti-PD-L1 mAb group compared to that in the control group (Fig. 4C). These results suggested that tumor antigen presentation and a certain degree of T cell priming had already occurred in lymph nodes at baseline prior to the anti-PD-L1 mAb treatment in the FM3A model but that PD-L1 on antigen-presenting cells suppressed T cell priming, and the anti-PD-L1 mAb unleashed the T cells (see hypothesized mechanism of action in Fig. 5G, left).

### Anti-PD-L1 mAb increased CXCR3^+^ activated T cells in lymph nodes, resulting in trafficking of T cells to tumors in response to CXCR3 ligands already expressed in tumors

Next we analyzed the time course of CD8^+^ T cell activation by anti-PD-L1 mAb treatment in lymph nodes and tumors in the FM3A model. Anti-PD-L1 mAb treatment increased the number of CD69^+^CD8α^+^ T cells in lymph nodes and the percentage of these cells in tumors on Day 8 (Fig. 5A). Importantly, on Day 4, anti-PD-L1 mAb treatment had increased these cells in lymph nodes but not in tumors (Fig. 5A). Additionally, anti-PD-L1 mAb treatment increased the numbers of CXCR3^+^CD69^+^CD8α^+^, CXCR3^+^Granzyme B (GzmB)^+^CD8α^+^, and CXCR3^+^Ki67^+^CD8α^+^ T cells in lymph nodes (Fig. 5B). FM3A tumors expressed CXCR3 ligands, especially CXCL9, regardless of anti-PD-L1 mAb treatment (Fig. 5C). CXCR3 mAb significantly prevented activated CD8α^+^ T cells from increasing in tumors, but not in lymph nodes, as a result of the anti-PD-L1 mAb treatment (Fig. 5D) and significantly attenuated the tumor growth inhibition of the anti-PD-L1 mAb (Fig. 5E). In contrast, in the OV2944-HM-1 model, which was insensitive to anti-PD-L1 mAb treatment, expression of CXCR3 ligands in the tumor was lower than that in the FM3A model and we confirmed that anti-PD-L1 mAb treatment increased the number of CD69^+^CD8α^+^ T cells in lymph nodes but not in tumors (Fig. 5F). These results indicated that the time lag between the anti-PD-L1 mAb-induced increase in activated CD8^+^ T cells in the lymph nodes and the increase in activated CD8^+^ T cells in tumors was caused by T cell trafficking from lymph nodes to tumors expressing CXCR3 ligands. These schemes are illustrated in Fig. 5G, right.

### Antitumor activity of anti-PD-L1 mAb predicted by expression of genes related to cross-presenting DCs and expression of CXCR3 ligands in tumor tissues

**Figure 6:**
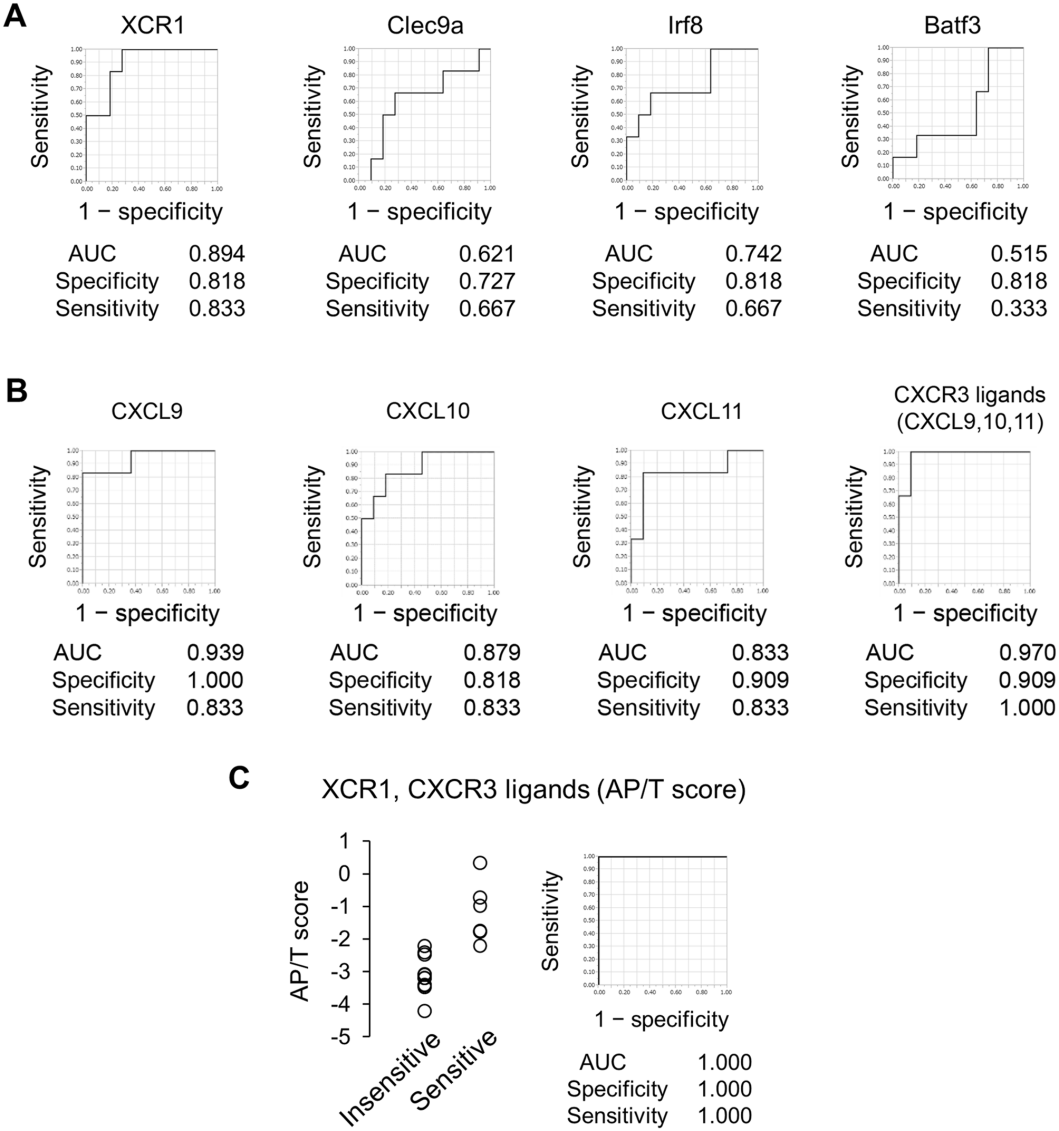
Relationship between antitumor activity of anti-PD-L1 mAb and antigen-presenting DC-related gene expression and/or gene expression of CXCR3 ligands in murine tumor models. (**A**) ROC curve analysis by expression of antigen-presenting DC-related genes in 17 murine tumor models. (**B**) ROC curve analysis by expression of CXCR3 ligands in 17 murine tumor models. (**C**) AP/T of anti-PD-L1 mAb-insensitive or -sensitive tumor tissues and ROC curve analysis.

Our data suggest that the necessary and sufficient conditions for efficacy of anti-PD-L1 antibody are not “pre-existing T cell” nor “PD-L1 expression in tumor” but the simultaneous presence of “T cell priming in lymph node” and “T cell recruitment capability (i.e., expression of CXCR3 ligands) in the tumors”. Considering that “T cell priming in lymph node” requires tumor-derived antigen presenting dendritic cells (APCs)^8^, intratumor gene expression of XCR1, Clec9a, Irf8, and Batf3, markers of APCs^9–12^, would be suitable markers that reflect the status of “T cell priming in lymph node”. To examine the ability of these APCs markers to classify tumors as anti-PD-L1 mAb-sensitive or -insensitive, we measured the mRNA of these markers in tumors of 17 murine tumor models. The AUC values of XCR1, Clec9a, Irf8, and Batf3 were 0.894, 0.621, 0.742, and 0.515, respectively (Fig. 6A). The specificity/sensitivity of these were 0.818/0.833, 0.727/0.667, 0.818/0.667, and 0.818/0.333, respectively.

Next, to examine the relation between the antitumor activity of anti-PD-L1 mAb and expression of CXCR3 ligands, we measured mRNA expressions of CXCL9, CXCL10, and CXCL11 in tumors, and analyzed the ROC curves. The AUC values of each of the three CXCR3 ligands as a single factor were 0.939, 0.879, and 0.833, respectively, and the AUC value obtained by using the average of the three was 0.970 (Fig. 6B). The specificity/sensitivity of the three CXCR3 ligands were 1.000/0.833, 0.818/0.833, and 0.909/0.833, respectively, and the specificity/sensitivity obtained by using the average of the three was 0.909/1.000. Thus, the AUC, sensitivity, and specificity of the three CXCR3 ligands were still less than perfect.

Because we supposed that sensitive tumors would have both (1) characteristic tumor antigen presentation in the tumor-draining lymph nodes and (2) characteristic expression of CXCR3 ligands in tumors at baseline, we created a combined biomarker—the Antigen-Presentation-related gene expression and T-cells-attracting-related gene expression combined biomarker (AP/T score)—defined as the averaged expression of XCR1 and CXCR3 ligands. The AUC of the AP/T score finally reached 1.000 with perfect sensitivity/specificity (Fig. 6C).

### Predictive ability of the AP/T score was also confirmed in OAK clinical study data

**Figure 7:**
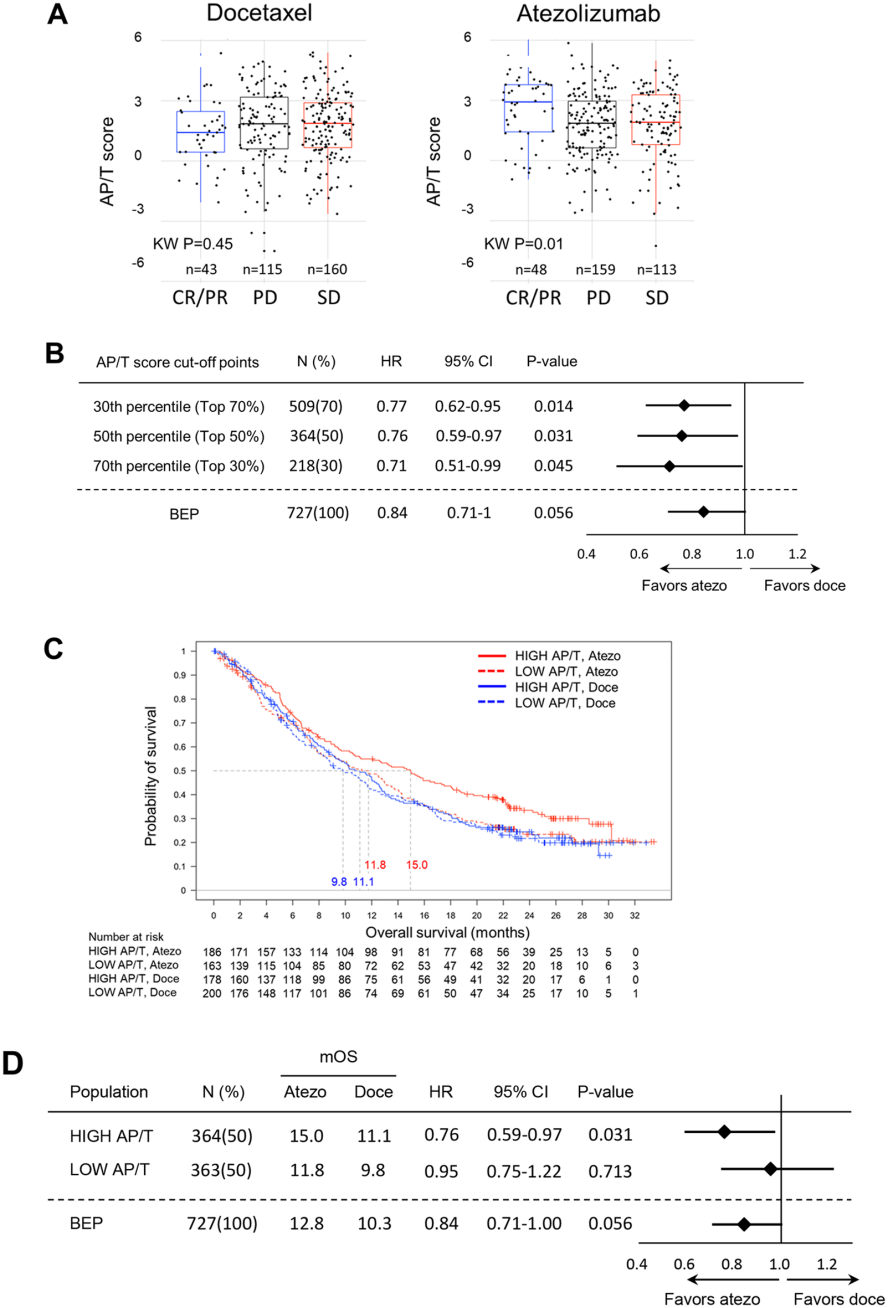
Association of clinical outcome and expression of antigen-presenting DC-related gene and CXCR3 ligands genes in tumor tissue in the phase 3 OAK trial. (**A**) Relation between overall response rate and AP/T score. (**B**) Forest plots of hazard ratios (HR) for OS in the biomarker-evaluable population (BEP) and in subgroups with AP/T scores above the 30th, 50th, and 70th percentile cut-off points (top 70%, 50%, and 30% of the population, respectively). (**C**, **D**) Kaplan–Meier estimates and forest plots of HRs for OS in the HIGH and LOW AP/T subgroups (cut at 50th percentile) in the atezolizumab and docetaxel treatment arms.

Having demonstrated that the AP/T score was associated with efficacy in the preclinical murine models, the usefulness of the AP/T score in predicting clinical efficacy was also tested by deriving it from gene expression data (mean Z of AP/T genes) obtained from tumor tissue from patients in the clinical phase 3 OAK trial in which atezolizumab improved OS regardless of PD-L1 expression (“Supplemental Figure”). The AP/T scores were higher in only atezolizumab-treated patients who achieved CP/PR (Fig. 7A), with no such trend seen in the docetaxel arm. Improved OS benefit was observed for the subgroups of patients with AP/T scores above all three cut-off points (≥ 30th, ≥ 50th, ≥ 70th percentile) relative to the biomarker-evaluable population (BEP) in the OAK study (Fig. 7B). In the subgroup with AP/T score ≥ 50th percentile cut-off point, the OS hazard ratio (HR) was 0.76 (95% CI 0.59–0.97; *P* = 0.031) numerically lower than that of the BEP and the median OS was 15.0 and 11.1 months in the atezolizumab and docetaxel treatment arms, respectively. Patients with high AP/T scores (≥ 50th percentile) obtained significant OS benefit from atezolizumab versus docetaxel, but no significant difference was observed (HR 0.95; 95% CI 0.75–1.22; P = 0.713) in the patients with low AP/T scores (< 50th percentile) (Fig. 7C,D).

## Discussion

### The role of PD-L1 in T cell priming and the mechanism of action of anti-PD-L1 mAb

We demonstrated that an anti-PD-L1 mAb exerted antitumor activity in a mouse tumor model that had little intratumoral expression of PD-L1 and little intratumoral infiltration of immune cells. PD-L1 blockade in tumor-draining lymph nodes appeared to trigger re-activation of T cells leading to antitumor activity (Fig. 5G).

It has been reported that PD-L1 is expressed on DCs in lymph nodes of patients with cancer^4^, and it has been recently reported that PD-L1 expressed on immune cells in addition to on tumor cells plays an essential role in PD-L1 blockade^13,14^. Our results showed that PD-L1 was highly expressed on CD103^+^CD11c^+^ DCs in tumor-draining lymph nodes, which are known to be specialized APCs capable of presenting tumor antigens to CD8^+^ T cells, and that the anti-PD-L1 mAb treatment enhanced T cell priming in the tumor-draining lymph nodes in a tumor antigen-specific manner. The depletion of not only CD8^+^ T cells but also CD4^+^ T cells completely abolished the antitumor activity of anti-PD-L1 mAb treatment, which may support the importance of the collaboration of CD4^+^ helper T cells and CD8^+^ T cells in the T cell priming necessary for the antitumor activity exerted by anti-PD-L1 mAb treatment in this model.

Taking these findings together, the current study suggests that PD-L1 blockade in tumor-draining lymph nodes is one of the possible mechanisms of action by which TC0 and IC0 tumors respond to anti-PD-L1 therapy in clinical studies. Interestingly, we found that the anti-PD-L1 mAb activated and increased the number of PD-1^−^B7-1^+^ T cells in lymph nodes. B7-1 expressed on activated T cells has also been reported to interact with PD-L1 causing negative regulation of effector T cell activation^2^. Anti-PD-L1 antibodies may block this interaction between B7-1 on T cells and PD-L1. Recently, however, it was proposed that interaction between PD-L1 and B7-1 can occur only *in cis* on the same cell in a manner that may block the binding of B7-1 to CD28 or PD-L1 to PD-1^15,16^. This indicates another possibility that anti-PD-L1 antibodies may block the interaction between B7-1 and PD-L1 on antigen-presenting cells and accelerate co-stimulation via binding between B7-1 on antigen-presenting cells and CD28 on T cells independent of PD-1 (Fig. 5G). Further study will be needed to clarify the precise role of PD-L1/B7-1 interactions in cancer immunity.

### Novel biomarker set predictive of anti-PD-L1 antibody efficacy

We demonstrated that PD-L1 blockade in tumor-draining lymph nodes was important for the efficacy of anti-PD-L1 mAb treatment. Many candidates have been investigated as biomarkers for predicting the efficacy of anti-PD-1 and anti-PD-L1 antibodies, including intratumoral PD-L1 expression, intratumoral T cell infiltration, and intratumoral interferon related gene signature^17–19^. All of those candidates were proposed on the basis of mechanisms of action only in the tumor site, and mechanisms of action in lymph nodes were not taken into account. In our study, to achieve a more precise level of predictability than that of conventional biomarkers, we additionally took into account the mechanism of action in tumor-draining lymph nodes. However, since direct detection of PD-L1 expression and T cell priming in lymph nodes is not clinically feasible, we needed to investigate other approaches to monitoring these factors in available specimens to enable more precise selection of potential responders.

Based on the cancer–immunity cycle, the release of tumor antigens and the presentation of the captured antigens on MHC molecules to T cells are essential in order for PD-L1 to have a role in T cell priming^8^. We found that lymphocytes taken from FM3A tumor-draining lymph nodes of control IgG-treated mice produced a considerable amount of IFNγ when co-cultured with FM3A cells, not by MHC-matched invalid MBT2, suggesting that tumor antigens have been captured by DCs and presented to specific CD8^+^ T cells in lymph nodes even at baseline in this tumors; however, due to PD-L1-mediated suppression, this may not be sufficient for optimal CD8^+^ T cell priming to control tumor growth. For CD8^+^ T cell priming, antigen cross-presenting DCs are the most important among the DC subpopulation. XCR1 is reported to be selectively expressed on DCs specialized in antigen cross-presentation in mice and potentially also in humans^20,21^. Since it has been well documented that intratumoral XCR1+/CD103+ play a critical role in antigen-presentation by engrafting tumor antigens and migrating to draining lymph nodes in CCR7-dependent homing^22^, the presence and/or increase of cross-presenting DCs in tumors may be a surrogate marker for T cell priming in tumor-draining lymph nodes. Importance of intratumor DC was also demonstrated by Spranger et al. that activation of tumor β-catenin signaling reduced intratumor CD103+ DC which resulted in defect in T cell priming in the tumor-draining lymph nodes^23^. Mayoux et al. have reported that the DCs gene signature dictate responses to PD-L1 blockade cancer immunotherapy^24^. However, we showed that the predictability using DSs gene signature alone was still less than perfect (Fig. 6B).

Furthermore, we focused on the fact that primed CD8^+^ T cells have to reach and infiltrate into the tumor in order to exert their antitumor activity. CXCR3 is a chemokine receptor that is highly expressed on primed effector T cells and plays an important role in T cell attraction and function^25,26^. We showed that anti-PD-L1 mAb treatment increased CXCR3^+^ effector CD8^+^ T cells in lymph nodes and that CXCR3 blockade abolished the antitumor activity of the anti-PD-L1 mAb treatment despite T cell activation in the lymph nodes. Chamoto et al. reported a similar phenomenon in a T-cell-inflamed Colon 38 tumor model^27^ suggesting the importance of this chemokine–receptor system also in tumors with an immune-inflamed phenotype. Chow et al. have reported that the CXCR3 chemokine system is a biomarker for sensitivity to PD-1 blockade^28^. However, we showed that the predictability using gene expression of CXCR3 ligands alone was still less than perfect (Fig. 6B).

Our results indicate that for antitumor activity to be exerted by anti-PD-L1 treatment, not only must the suppressed T cell priming be released but also a tumor environment necessary to attract activated T cells from the blood must be present at baseline. Therefore, we conducted a combined analysis (the AP/T score) involving expression of genes related to antigen presentation (XCR1) and expression of genes related to T cell attraction (CXCR3 ligands). ROC curves are frequently used for assessment of predictability of biomarkers. The AUC of this AP/T score was 1, which means that there was a cut-off point that could completely identify only sensitive tumors which include both PD-L1-positive/inflamed tumors (e.g. colon 38) and PD-L1-negative/non-inflamed tumors (e.g. FM3A) among the 17 mouse tumor models examined. Furthermore, using the gene expression data from patients in the OAK trial in which a survival benefit was observed even in the subgroup with TC0/IC0, we demonstrated that the AP/T score is a predictive biomarker for OS in patients with NSCLC treated with atezolizumab monotherapy. At increasing AP/T cut-offs, ranging from the 30th percentile to the 70th percentile, the hazard ratio for OS improved from 0.77 (≥ 30th percentile [top 70% of the population]) to 0.71 (≥ 70th percentile [top 30% of the population]), emphasizing the positive correlation between increasing AP/T score and improved clinical outcomes.

Despite the success of this AP/T score in predicting the efficacy of anti-PD-L1 treatment, we consider that the AP/T score comprising the combination of the expression of XCR1 and CXCR3 ligands is one representative example and that this particular combination can be modified. For example, tumor mutational burden and mismatch repair status are considered to be indicators of the potential immunogenicity of tumors and have been tested as biomarkers for atezolizumab^29^. These markers may become a substitute or a supplement for tumor XCR1 expression as the tumor antigen-presentation-related marker. Indeed, patients with no or low PD-L1 expression (TC0 and IC0) and a high blood-based tumor mutational burden score showed a trend towards a progression-free survival benefit^29^, and a patient with mismatch repair-deficient hepatoid adenocarcinoma of the lung was reported to respond to anti-PD-L1 therapy despite there being no PD-L1 expression in the tumor^30^. Furthermore, with regard to the marker for T cell attraction, chemokine receptor CCR5 is also involved in the migration of tumor-specific T cells toward sites of autologous tumors^31^. CCR5 ligands may become a substitute or a supplement for CXCR3 ligands in terms of gene expression related to the attraction of activated T cells. In fact, the combination of blood-based tumor mutational burden and multiple chemokines may also be useful for predicting the efficacy as a substitute for the combination of XCR1 and CXCR3 ligands in the tumor. What is important is that these are not sufficient if each is applied alone. In other words, it is the combination of antigen-presentation and effector T cell recruitment capacities that is a critical for precisely predicting responders. Furthermore, this concept covers both inflamed tumors and PD-L1-negative immune-desert tumors.

In conclusion, we have shown that an anti-PD-L1 mAb exerted its antitumor activity through the blocking of PD-L1 in tumor-draining lymph nodes as well as at the tumor site, which would provide a scientific rationale for the efficacy of PD-L1 antibodies in patients with little intratumoral PD-L1 expression. Furthermore, we demonstrated that a novel biomarker combining tumor antigen-presentation-related gene expression and activated T cell attraction-related gene expression predicts the response to atezolizumab. These data suggest that this novel approach—putting more emphasis on activities in tumor-draining lymph nodes—will enable us to develop a predictive biomarker for anti-PD-L1 antibodies such as atezolizumab, and this concept may extend further and be applicable to other immune checkpoint inhibitors.

## Materials and methods

### Cell lines and culture conditions

We used 17 different cancer cell lines (suppliers and culture conditions are listed in Supplementary Table S2). All cells were maintained at 37 °C under 5% CO_2_. CMT64-OVA cells were CMT64 cells transfected by lipofection with the plasmid vector encoding neo-OVA which carries a complete copy of chicken ovalbumin (OVA) mRNA and the neomycin (G418) resistance gene.

### Mice

Female 5- to 8-week-old C3H/He, C57BL/6, B6C3F1 (C57BL/6 × C3H/He), BALB/c, and DBA/2 mice were obtained from Charles River Japan (Kanagawa, Japan) and CLEA Japan (Tokyo, Japan). All animals were allowed to acclimatize and recover from shipping-related stress for 1 week prior to the study. The animals were allowed free access to chlorinated water and irradiated food, and were kept in a controlled light–dark cycle (12–12 h)^32^.

### Tumor models

Colon 38 cells were resuspended in a 50:50 mixture of growth factor-reduced Matrigel (BD Biosciences, Franklin Lakes, NJ, USA) and culture medium without fetal bovine serum (FBS), and 5.0 × 10^6^ cells were implanted subcutaneously into the right flank of C57BL/6 mice. All other tumor cells were resuspended in culture medium without FBS, and each mouse was subcutaneously injected with 1.0 × 10^6^ cells as follows. Hepa 1–6, EL4, B16F1, B16F10, LLC1, CMT64, and CMT64-OVA cells were implanted into the right flank of C57BL/6 mice. MH134-TC, MBT2, and FM3A cells were implanted into the right flank of C3H/He mice. EMT6, Renca, MethA, and 4T1 cells were implanted into the right flank of BALB/c mice. OV2944-HM-1 cells were implanted into the right flank of B6C3F1 mice. KLN 205 cells were implanted into the right flank of DBA/2 mice.

When tumor volumes reached approximately 50–200 mm^3^, mice were randomly allocated to each cage corresponding to each treatment group and administration of antibody was started (Day 1). Anti-mouse PD-L1 mAb (10F.9G2; BioLegend, Diego, CA, USA) or Rat IgG (MP Biomedicals, Santa Ana, CA, USA) was administered intraperitoneally to the mice at a dose of 10 mg/kg three times a week. Anti-mouse CXCR3 mAb (CXCR3-173; BioLegend) or Hamster IgG (SHG-1; BioLegend) was administered intraperitoneally at a dose of 50 µg/head twice a week. For CD8^+^ cell or CD4^+^ cell depletion experiments, mice bearing FM3A were injected with anti-mouse CD8α mAb (53–6.7; BioLegend) or anti-mouse CD4 mAb (GK1.5; BioXcell, West Lebanon, NH, USA) at 200 µg/head on Day-4 and thereafter twice a week. The efficacy results of these drugs have been confirmed to be similar in multiple studies with different technical assistants and investigators, blinding was not performed after randomization.

### Flow cytometric analysis^32^

For analysis of tumor-infiltrating lymphocytes, tumor tissue was excised from control-treated mice and anticancer agent-treated mice, and single-cell suspensions were obtained by mincing tumors and homogenizing them by disruption and digestion with a gentleMACS Dissociator and a Tumor Dissociation Kit for mice (Miltenyi Biotec, Bergisch Gladbach, Germany). For analysis of lymph nodes, lymphocytes from axillary and brachial lymph nodes on the right side of tumor-bearing mice were harvested and mixed. For analysis of peripheral blood, blood was pretreated with VersaLyse lysing solution for red blood cell lysis (Beckman Coulter, Brea, CA, USA). Single-cell suspensions were incubated with anti-Fcγ receptor antibodies (Tonbo Biosciences, San Diego, CA, USA) and the fixable viability dye FVD506 or FVD780 (eBioscience, San Diego, CA, USA) at 4 °C for 10 min, and stained with the following monoclonal antibodies: mouse CD45 (30-F11), CD4 (RM4-5), CD8α (53–6.7), CD69 (H1.2F3), CD11c (HL3), CD103 (M290), CXCR3 (CXCR3-173), PD-L1 (MIH5), F4/80 (T45-2342), CD44 (IM7), CD62L (MEL-14), B7-1 (16-10A1), PD-1 (J43), Foxp3 (FJK-16s), Granzyme B (GB11), and Ki67 (B56) from BioLegend or BD Biosciences. The appropriate conjugated isotype-matched immunoglobulin G (IgG) was used as the control for each. Intracellular cytokine staining was performed with the use of a Foxp3/Transcription Factor Staining Buffer Set (eBioscience). Cells were analyzed using an LSRFortessa X-20 cell analyzer (BD Biosciences) and FlowJo 10 software (Tree Star, San Carlos, CA, USA).

### Tumor-stimulated IFNγ release assay^32^

Tumor-draining lymph nodes from the FM3A model were assessed on Day 7 for specific antitumor response by analyzing IFNγ release. Whole lymphocytes from axillary, brachial, and inguinal lymph nodes on the right side of tumor-bearing mice were harvested and mixed. These lymphocytes were co-cultured with irradiated tumor cells (100 Gy) in a 5:1 ratio (lymphocytes/tumor cells) at 37 °C for 3 days. FM3A cells were the target cells, and MBT2 cells were used as a negative control. IFNγ in the culture supernatant was examined by ELISA (R&D Systems, Minneapolis, MN, USA).

### TCRα repertoire analysis

For analysis of T cell receptors (TCR) on tumor-infiltrating lymphocytes, tumor tissue was excised from control-treated mice and anti-PD-L1 mAb-treated mice, and was then suspended in RNAlater Stabilization Solution (Thermo Fisher Scientific). Purification of RNA and analysis by next-generation sequencing was conducted by Repertoire Genesis (Ibaraki, Japan). The Simpson diversity index was used to compare diversities of TCRα repertoires^33^.

### RNA sequencing

Tumor tissues were collected after the tumors were established in the immunocompetent mice. Total RNA was isolated from tumor tissue by using a NucleoSpin RNA Kit (Macherey–Nagel, Düren, Germany) according to the manufacturer’s instructions. RNA quality control was performed with the 2200 TapeStation (Agilent-Technologies, Santa Clara, CA, USA). RNA-seq libraries were prepared by using a TruSeq RNA Library Prep kit v2 (Illumina, San Diego, CA, USA) according to the manufacturer’s instructions. Sequencing was performed using the Illumina HiSeq 2500 Sequencing System (100-bp paired-end sequencing). RNA log expression (*R*) was estimated from the equations *R* = log (FPKM value for target mRNA) − log (FPKM value for housekeeping gene).

### Antigen-presentation-related gene expression and T-cells-attraction-related gene expression combined score (AP/T score)

AP/T score was calculated as follows:

$$ {\text{AP}}/{\text{T score}} = \left\{ {R\left( {{\text{XCR1}}} \right) + R\left( {{\text{CXCL9}}} \right) + R\left( {{\text{CXCL1}}0} \right) + R\left( {{\text{CXCL11}}} \right)} \right\}/{\text{4}} $$

### Immunohistochemistry

We evaluated the localization of CD8α^+^ T cells in tumor tissue by immunohistochemical staining of CD8α (rat anti-mouse CD8α mAb, KT15 [GeneTex, Irvine, CA, USA]). We evaluated the expression of PD-L1 in tumor tissue by immunohistochemical staining of PD-L1 (anti-mouse B7-H1/PD-L1 polyclonal goat antibody [R&D Systems]).

### Immunoassays

After mice were euthanized by exsanguination on Day 8, tumors were collected and homogenized. Concentrations of mouse CXCL9 were measured with a Quantikine ELISA kit (R&D Systems). CXCL10 was quantified by using a mouse CXCL10 ELISA kit (Thermo Fisher Scientific). CXCL11 was quantified by using a specific ELISA kit (Cusabio Biotech, Selangor, Malaysia).

### Clinical study design and assessments^3^

The OAK trial (NCT02008227) was a randomized phase 3 study that compared atezolizumab with docetaxel in metastatic NSCLC. Patients with NSCLC with measurable disease and previous chemotherapy were randomized 1:1 to intravenous atezolizumab at 1200 mg every 3 weeks or intravenous docetaxel at 75 mg/m^2^ every 3 weeks. The primary end point was OS. Atezolizumab improved OS compared to docetaxel, which led to FDA approval of atezolizumab (also known as Tecentriq) for patients with advanced NSCLC who have disease progression during or following platinum-based chemotherapy. For transcriptional profiling of tumors with RNA-sequencing TruSeq RNA Access technology (Illumina^®^) was used. RNA-sequencing data were generated from pre-treatment FFPE tumor tissue samples.

### Statistical analysis^32^

Analysis of AP/T score was performed retrospectively. Overall response rate was assessed by the investigator using RECIST v1.1. OS is defined as the time between the date of randomization until death due to any cause. Treatment arms were compared for OS individually using a univariate Cox proportional hazards model without stratification in the BEPs and their subgroups. No multiplicity correction was applied to *P* values or to 95% CIs. All *P* values are two sided. *P* < 0.05 was considered to indicate a significant difference. Kaplan–Meier methodology was used to estimate the median OS and to construct the survival curves. Gene expression levels between the CR/PR group, SD group, and PD group were compared with a Kruskal–Wallis test.

To evaluate statistical significance in mouse model experiments, data was analyzed with the Wilcoxon test. For two groups, *P* < 0.05 was considered to indicate a significant difference. For multiple groups, *P* values were adjusted by the Holm − Bonferroni method^34^ by using JMP version 10 software (SAS Institute, Cary, NC, USA). Construction of the receiver operating characteristic (ROC) curves was performed using JMP version 10 software, and the areas under the ROC curves (AUC) were calculated to evaluate the diagnostic accuracy and to compare AUC for all tested parameters separately. Tumor volume (*V*) was estimated from the equation *V* = *L* × *W*^2^ × 0.5 (*L* = length; *W* = width). The percentage of tumor growth inhibition (TGI%) was calculated as follows:

$$ \begin{aligned}   {\text{TGI}}\%  &  = \{ 1 - ({\text{tumor volume of treatment group on}} \\     & \quad {\text{final evaluation day}} - {\text{tumor volume of treatment group}} \\     & \quad {\text{on Day }}1)/({\text{tumor volume of control group at final}} \\     & \quad {\text{evaluation day}} - {\text{tumor volume of control group on Day}} \\     & \quad 1) \times 100. \\  \end{aligned} $$

### Ethical approval

All animal procedures were approved by the Institutional Animal Care and Use Committee at Chugai Pharmaceutical Co., Ltd., and conformed to the *Guide for the Care and Use of Laboratory Animals* published by the Institute of Laboratory Animal Resources (ILAR). The study was carried out in compliance with the ARRIVE guidelines. The OAK study was done in full accordance with the guidelines for Good Clinical Practice and the Declaration of Helsinki, and all patients gave written informed consent. We performed the human investigations after approval by the research ethics committee of Chugai Pharmaceutical Co., Ltd.

## Supplementary Information

Supplementary Information.

## Abbreviations

APC: Antigen cross-presenting cell
AUC: Area under ROC curve
DCs: Dendritic cells
FPKM: Fragments per kilobase of exon per million mapped fragments
IgG: Immunoglobulin G
mAb: Monoclonal antibody
PD-1: Programmed cell death 1
PD-L1: Programmed death ligand 1
ROC curve: Receiver operating characteristic curve
TC0/IC0: Low or undetectable PD-L1 on tumor cells and immune cells

## References

[1] Zou W, Wolchok JD, Chen L (2016). PD-L1 (B7–H1) and PD-1 pathway blockade for cancer therapy: Mechanisms, response biomarkers, and combinations. Sci. Trans. Med..

[2] Butte MJ, Keir ME, Phamduy TB, Sharpe AH, Freeman GJ (2007). Programmed death-1 ligand 1 interacts specifically with the B7-1 costimulatory molecule to inhibit T cell responses. Immunity.

[3] Rittmeyer A (2017). Atezolizumab versus docetaxel in patients with previously treated non-small-cell lung cancer (OAK): A phase 3, open-label, multicentre randomised controlled trial. Lancet (London, England).

[4] Curiel TJ (2003). Blockade of B7-H1 improves myeloid dendritic cell-mediated antitumor immunity. Nat. Med..

[5] Gros A (2014). PD-1 identifies the patient-specific CD8(+) tumor-reactive repertoire infiltrating human tumors. J. Clin. Investig..

[6] van de Ven R (2017). High PD-1 expression on regulatory and effector T-cells in lung cancer draining lymph nodes. ERJ Open Res..

[7] Butte MJ, Pena-Cruz V, Kim MJ, Freeman GJ, Sharpe AH (2008). Interaction of human PD-L1 and B7–1. Mol. Immunol..

[8] Chen DS, Mellman I (2013). Oncology meets immunology: The cancer–immunity cycle. Immunity.

[9] Hildner K (2008). Batf3 deficiency reveals a critical role for CD8alpha+ dendritic cells in cytotoxic T cell immunity. Science (New York, NY).

[10] Piva L (2012). Cutting edge: Clec9A+ dendritic cells mediate the development of experimental cerebral malaria. J. Immunol. (Baltimore, Md.: 1950).

[11] Tailor P, Tamura T, Morse HC, Ozato K (2008). The BXH2 mutation in IRF8 differentially impairs dendritic cell subset development in the mouse. Blood.

[12] Yamazaki C (2013). Critical roles of a dendritic cell subset expressing a chemokine receptor, XCR1. J. Immunol. (Baltimore, Md.: 1950).

[13] Lau J (2017). Tumour and host cell PD-L1 is required to mediate suppression of anti-tumour immunity in mice. Nat. Commun..

[14] Tang H (2018). PD-L1 on host cells is essential for PD-L1 blockade-mediated tumor regression. J. Clin. Investig..

[15] Chaudhri A (2018). PD-L1 binds to B7-1 only *in cis* on the same cell surface. Cancer Immunol. Res..

[16] Sugiura D (2019). Restriction of PD-1 function by *cis*-PD-L1/CD80 interactions is required for optimal T cell responses. Science (New York, NY).

[17] Fehrenbacher L (2018). Updated efficacy analysis including secondary population results for OAK: A randomized phase III study of atezolizumab versus docetaxel in patients with previously treated advanced non-small cell lung cancer. J. Thorac. Oncol. Off. Publ. Int. Assoc. Study Lung Cancer.

[18] Herbst RS (2014). Predictive correlates of response to the anti-PD-L1 antibody MPDL3280A in cancer patients. Nature.

[19] Kowanetz M (2017). MA 05.09 pre-existing immunity measured by Teff gene expression in tumor tissue is associated with atezolizumad efficacy in NSCLC. J. Thorac. Oncol..

[20] Bachem A (2012). Expression of XCR1 characterizes the Batf3-dependent lineage of dendritic cells capable of antigen cross-presentation. Front. Immunol..

[21] Lei Y, Takahama Y (2012). XCL1 and XCR1 in the immune system. Microbes Infect..

[22] Roberts EW (2016). Critical role for CD103(+)/CD141(+) dendritic cells bearing CCR7 for tumor antigen trafficking and priming of T cell immunity in melanoma. Cancer Cell.

[23] Spranger S, Bao R, Gajewski TF (2015). Melanoma-intrinsic β-catenin signalling prevents anti-tumour immunity. Nature.

[24] Mayoux M (2020). Dendritic cells dictate responses to PD-L1 blockade cancer immunotherapy. Sci. Transl. Med..

[25] Groom JR, Luster AD (2011). CXCR3 in T cell function. Exp. Cell Res..

[26] Shimizu K (2016). Systemic DC activation modulates the tumor microenvironment and shapes the long-lived tumor-specific memory mediated by CD8^+^ T Cells. Can. Res..

[27] Chamoto K (2017). Mitochondrial activation chemicals synergize with surface receptor PD-1 blockade for T cell-dependent antitumor activity. Proc. Natl. Acad. Sci. USA.

[28] Chow MT (2019). Intratumoral activity of the CXCR3 chemokine system is required for the efficacy of anti-PD-1 therapy. Immunity.

[29] Gandara DR (2018). Blood-based tumor mutational burden as a predictor of clinical benefit in non-small-cell lung cancer patients treated with atezolizumab. Nat. Med..

[30] Basse V (2018). A mismatch repair-deficient hepatoid adenocarcinoma of the lung responding to anti-PD-L1 durvalumab therapy despite no PD-L1 expression. J. Thorac. Oncol. Off. Publ. Int. Assoc. Study Lung Cancer.

[31] Franciszkiewicz K (2009). Intratumoral induction of CD103 triggers tumor-specific CTL function and CCR5-dependent T-cell retention. Cancer Res..

[32] Iwai, T. *et al.* Topoisomerase I inhibitor, irinotecan, depletes regulatory T cells and up-regulates MHC class I and PD-L1 expression, resulting in a supra-additive antitumor effect when combined with anti-PD-L1 antibodies. *Oncotarget* **9**, 31411–31421. 10.18632/oncotarget.25830 (2018). 10.18632/oncotarget.25830 PMC6101148 30140379

[33] Venturi V, Kedzierska K, Turner SJ, Doherty PC, Davenport MP (2007). Methods for comparing the diversity of samples of the T cell receptor repertoire. J. Immunol. Methods.

[34] Holm S (1979). A simple sequentially rejective multiple test procedure. Scand. J. Stat..

