# Supplementary material for: Both T cell priming in lymph node and CXCR3-dependent migration are the key events for predicting the response of atezolizumab
Source: Sci Rep. 2021 Jul 6;11:13912. doi: 10.1038/s41598-021-93113-y (PMC8260627; doi:10.1038/s41598-021-93113-y)
Supplement: Supplementary file 1 — Supplementary Information. [file 41598_2021_93113_MOESM1_ESM.pdf]

Research Articles:

Title:

Importance of lymph nodes as a site of action of anti-PD-L1 antibodies, leading to identification of novel predictive biomarkers beyond PD-L1 expression score

Authors:

Toshiki Iwai[1], Masamichi Sugimoto[1], Namrata S. Patil[2], Daniel Bower[2], Miho Suzuki[1], Chie Kato[3], Keigo Yorozu[1], Mitsue Kurasawa[1], David S. Shames[2], and Osamu Kondoh[1]

Supplementary Data

Supplemental Fig.

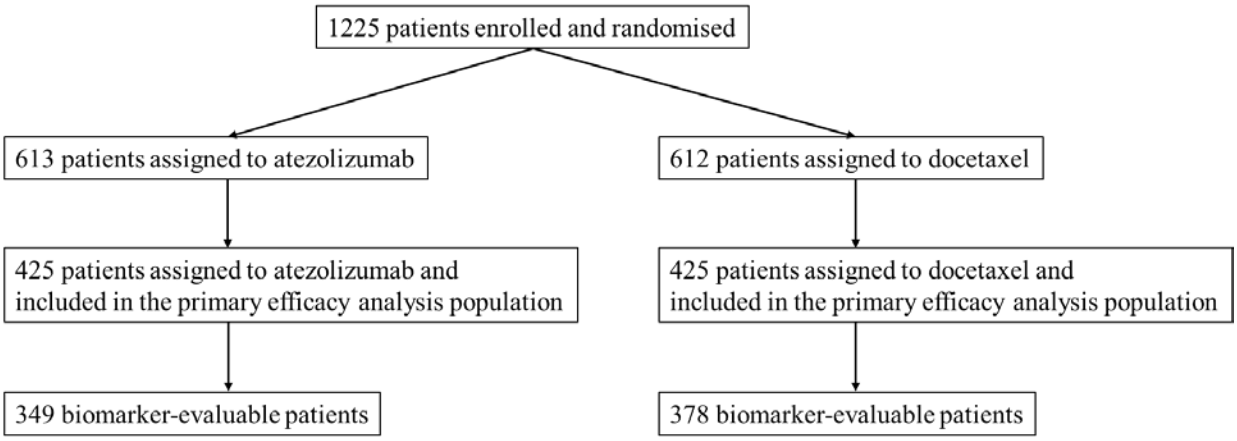

Supplemental Figure. CONSORT (Consolidated Standards of Reporting Trials) diagram for biomarker analysis from the OAK trial of atezolizumab in patients with previously treated, locally advanced, or metastatic NSCLC

Table S1. Antitumor activity of anti-PD-L1 mAb in the murine syngeneic tumor models

**Cell line**	**TGI (%)**	**Response**
Hepa 1-6	119	
Colon 38	90	
EMT6	87	Sensitive
MH134-TC	80	*P* < 0.05
MBT2	58	
FM3A	57	
OV2944-HM-1	—	
Renca	—	
MethA	—	
EL4	—	
KLN 205	—	Insensitive
B16F1	—	*P* > 0.05
B16F10	—	
LLC1	—	
4T1	—	
CMT64	—	
CMT64-OVA	—	

TGI, tumor growth inhibition

Table S2. Cell lines and culture conditions

**Cell lines**	**Suppliers**	**Culture conditions**
Hepa 1-6	ATCC	D-MEM with 10% FBS
Colon 38	JFCR	RPMI-1640 with 10% FBS
EMT6	ATCC	Waymouth MB 752/1 with 15% FBS
MH134-TC	IDAC	RPMI-1640 with 10% FBS
MBT2	JCRB Cell Bank	E-MEM with 10% FBS
FM3A	RIKEN BRC	RPMI-1640 with 10% Newborn Calf Serum
OV2944-HM-1	RIKEN BRC	α-MEM with 10% FBS
Renca	ATCC	RPMI-1640 with 10% FBS, 0.25% D-glucose, 0.1 mM NEAA, 10 mM HEPES, 2 mM L-glutamine, and 1 mM sodium pyruvate
MethA	JFCR	Ascites forms of MethA were propagated intraperitoneally in BALB/c mice
EL4	ATCC	RPMI-1640 with 10% FBS, 2.5 g/L D-glucose, 1 mM sodium pyruvate, 10 mM HEPES, and 50 µM 2-mercaptoethanol
KLN 205	ATCC	E-MEM with 10% FBS, 1 mM sodium pyruvate, 0.1 mM NEAA, and 1.5 g/L sodium bicarbonate
B16F1	ATCC	D-MEM with 10% FBS
B16F10	IDAC	RPMI-1640 with 10% FBS
LLC1	ATCC	D-MEM with 10% FBS
4T1	ATCC	RPMI-1640 with 10% FBS, 10 mM HEPES, 1 mM sodium pyruvate, and 0.45% D-glucose
CMT64	Public Health England	D-MEM with 10% FBS
CMT64-OVA	—	D-MEM with 10% FBS and 500 µg/mL G418

ATCC, American Type Culture Collection

JFCR, Japanese Foundation for Cancer Research

IDAC, Institute of Development, Aging and Cancer, Tohoku University

RIKEN BRC, RIKEN BioResource Research Center
